# AI in Cardiac Imaging: A UK-Based Perspective on Addressing the Ethical, Social, and Political Challenges

**DOI:** 10.3389/fcvm.2020.00054

**Published:** 2020-04-15

**Authors:** Matthew E. Fenech, Olly Buston

**Affiliations:** Future Advocacy, London, United Kingdom

**Keywords:** artificial intelligence, ethics, policy, principles, regulation

## Abstract

Imaging and cardiology are the healthcare domains which have seen the greatest number of FDA approvals for novel data-driven technologies, such as artificial intelligence, in recent years. The increasing use of such data-driven technologies in healthcare is presenting a series of important challenges to healthcare practitioners, policymakers, and patients. In this paper, we review ten ethical, social, and political challenges raised by these technologies. These range from relatively pragmatic concerns about data acquisition to potentially more abstract issues around how these technologies will impact the relationships between practitioners and their patients, and between healthcare providers themselves. We describe what is being done in the United Kingdom to identify the principles that should guide AI development for health applications, as well as more recent efforts to convert adherence to these principles into more practical policy. We also consider the approaches being taken by healthcare organizations and regulators in the European Union, the United States, and other countries. Finally, we discuss ways by which researchers and frontline clinicians, in cardiac imaging and more broadly, can ensure that these technologies are acceptable to their patients.

## Introduction

Technological change is certainly not a new phenomenon. 3.3 million-year old stone tools made by Australopithecus, one of the earliest hominid species, have been found in Kenya (1), indicating that the drive to use tools to make tasks easier, and hence to improve quality of life, has not changed over the millions of years of humanity's history. One thing that has certainly changed over this time period, however, is the increased speed at which technological progress occurs. Gordon Moore's famous prediction that the number of transistors per square inch on an integrated circuit would double every one to two years (2) has stood the test of time, and other metrics of technological advancement, such as data storage per unit cost, speed of DNA sequencing, and internet bandwidth, have also increased at exponential rates over the last few decades (3).

With new technologies come new potential socioeconomic impacts, and new reactions to these real and imagined impacts by governments and international bodies. Once again, the impulse to regulate novel technologies is long-standing—the history of everything from the railways, to the automobile, to mining, to *in vitro* fertilization provides fascinating case studies in how societies of the day reacted to unfamiliar technology. Nevertheless, it is arguable that artificial intelligence (AI) is unlike other technologies in that never before has there been such a general-purpose technology that makes us question what it means to be human (4–6). Although there is no sign of anything approaching artificial general intelligence (AGI), the very fact that AI poses such deeply existential questions is just one of the challenges that it poses to regulators and policymakers, particularly in the hugely sensitive area of healthcare. In this paper, we discuss the various ethical, social, and political challenges the application of AI to health and care presents, and how reactions to these challenges are being used to develop principles for action. In some jurisdictions, these principles are being translated into policy and regulation, clearly setting out what should and should not be allowed. Moreover, we outline what researchers and clinicians can do to help ensure that the use of these technologies is acceptable to patients and practitioners alike.

## Ethical, Social, And Political Challenges

Future Advocacy, an independent think tank focused on policy development around the responsible use of emerging technology, conducted a series of interviews with expert clinicians, technologists, and ethicists, as well as focus groups with patients, and identified ten sets of questions that are raised by the application of AI to the health setting (Table 1) (7). In the following sections, we briefly discuss each in turn, and reflect on any advances in thinking and practice that have occurred since the publication of our original report.

**Table 1:** Ten major ethical, social, and political challenges of the use of artificial intelligence technologies in health and care.

1. What effect will AI have on human relationships in health and care?
2. How is the use, storage and sharing of medical data impacted by AI?
3. What are the implications of issues around algorithmic transparency/explainability on health?
4. Will these technologies help eradicate or exacerbate existing health inequalities?
5. What is the difference between an algorithmic decision and a human decision?
6. What do patients and members of the public want from AI and related technologies?
7. How should these technologies be regulated?
8. Just because these technologies could enable access to new information, should we always use them?
9. What makes algorithms, and the entities that create them, trustworthy?
10. What are the implications of collaboration between public and private sector organizations in the the development of these tools?

### Relationships

Healthcare is built on a complex network of relationships between various stakeholders. The primacy of the relationship between patients and their healthcare professionals (HCPs) is clear from the value still placed on it, even in the context of medical practice that is increasingly characterized by the use of technology (5, 7). It is however but one of the relationships in healthcare—others include those between the HCP and caregivers/relatives; between different HCPs; between top-level administrators and HCPs “on the ground”; and between patients and wider society (8). All of these relationships could be impacted by the introduction of an AI algorithm, and the inferences or predictions it provides, as a “third party” in what were previously two-way interactions. What will patients do, for example, when faced with the scenario of their doctor's recommendation clashing with the suggestion for treatment provided by an AI tool? How will patients react to an error in their care that is traced back to a decision made, or supported, by AI? The specific issue of liability for error is discussed in section 2.5 below, and a Royal Society-commissioned study found that many members of the public were optimistic about the possibility for AI to *reduce* medical error (5), but there is a need for more research aimed at understanding how patients are likely to respond to such AI-derived errors.

Another way by which AI may impact relationships in healthcare is through its potential to fundamentally change the role of doctors and other HCPs. To paraphrase Mark Twain, reports of the death of the radiologist are greatly exaggerated (9). Nevertheless, as AI tools become better at performing certain circumscribed tasks in healthcare, such as image recognition, the repertoire of tasks that make up a HCP's job will change. Some have expressed their hope that the “delegation” of such tasks to algorithms will free up more time for HCPs to spend with patients and their relatives (10), but previous experience of the introduction of different technologies into the clinical space suggests that they may well increase clinician workload in both primary and secondary care (11–13). Whether AI is different remains unknown; various medical bodies are grappling with this question (14, 15), and at the time of writing, Health Education England (the body in England responsible for postgraduate training and development of NHS England's workforce) was holding a consultation on the topic of the “Future Doctor” (16).

### Data

AI is increasingly being used to identify patterns in and extract value from the vast amounts of data being generated by individuals, governments, and companies. Healthcare is no exception—the volume, complexity and longevity of healthcare data are all rising fast, with some estimates predicting that the total amount of healthcare data will reach 2.3 billion gigabytes by next year (17). With larger volumes and greater complexity come new questions about the implications of such data use and storage. Firstly, there is the pragmatic concern of how informed consent—the bedrock of interactions between patients and healthcare systems since at least the nineteenth century (18)—is obtained from each and every contributor to a dataset, which may number in the millions. Similarly, as the technology is developing so rapidly, new insights are derived from existing datasets that could not have been predicted before data analysis, as evidenced by the Google/Verily Life Science deep learning algorithm that can determine gender from retinal photographs (19). How do we obtain informed consent for future, unimagined uses of data? The European Union (EU) General Data Protection Regulation (GDPR) already makes it clear that there are multiple “lawful bases” for data processing, and informed consent is only one of them (20). Clearly, the field of health and care needs to determine whether alternatives to individualized informed consent (including broad consent, “opt-out” consent, and presumed consent) are acceptable in the context of AI research and development, whilst maintaining their patients' and research subjects' trust (21). GDPR also sets clear restrictions on how identifiable information about particular data subjects can or cannot be shared, and these are particularly relevant in the age of establishment and curation of “big data” healthcare datasets. Patients and research subjects are right to expect that their data, donated in good faith for use in research, does not end up being used to determine health insurance premiums, for example (22). Although the regulation exists, this is only as good as its enforcement, and concerns about the rigor with which GDPR is being enforced have been raised in other sectors (23). Ultimately, when it comes to such sensitive subjects, a reliance on regulation alone is not sufficient; this must be backed up by education of, and dialogue between, all stakeholders, focusing on their data rights and responsibilities in law.

A consideration that is perhaps particularly relevant to radiologists was highlighted in the Joint Statement on “Ethics of Artificial Intelligence in Radiology,” issued by the American College of Radiologists, European Society of Radiology, Radiological Society of North America, Society for Imaging Informatics in Medicine, European Society of Medical Imaging Informatics, Canadian Association of Radiologists, and American Association of Physicists in Medicine (24). Radiologists are in great demand to provide accurate and replicable labeling of radiological images, which are then used in supervised learning, for example in training convolutional neural networks. Those with expertise in cardiac imaging will be particularly sought after, given the especially time-consuming and resource-intensive nature of interpreting cardiac imaging modalities such cardiac magnetic resonance (25), and any difficulty in recruiting such experts may well slow the development of these tools in this area of radiology. As any practicing clinician knows, labeling and classification of real-world clinical imaging is similar to all medical decision-making in that it involves many assumptions, heuristics, and potential biases (26–28). When processing data for use in AI training, radiologists need to be aware of these biases, to avoid introducing additional bias into imperfect datasets, as well as recognizing the various incentives and pressures that may influence their decision-making, including commercial pressures to provide these data (24, 29, 30). Radiology training programmes will need to be updated to make sure the radiologists of the future are best prepared to spot and mitigate these problems (31).

Perhaps of all the challenges discussed in this review, those surrounding data are the ones best addressed by existing regulation, with the Privacy Rule created under the Health Insurance Portability and Accountability Act (HIPAA) well-established in the United States, and GDPR incentivizing businesses and public bodies to give their European clients greater control of their data (32). Nevertheless, gaps remain. HIPAA's Privacy Rule, for example, does not cover non-health information from which health-related conclusions can be drawn, or user-generated health information (33)—such omissions cannot be tolerated for long in an age of linked datasets and wearable technologies constantly monitoring parameters such as heart rate. Moreover, although Article 22 on automated decision-making is clearly relevant, the words “artificial intelligence” do not appear in the text of the GDPR once, as the regulation is relatively agnostic about the downstream use of the data. This is in contrast to, for example, the guidance on the regulation of data-driven technology published by the German Government's Data Ethics Commission, which explicitly draws links between data ethics and algorithmic ethics (34). European policy experts have reason to believe that this document will prove influential as the European Commission (EC) develops widely-expected regulation on AI in 2020 (35). The framework for such regulation was laid out in the EC's White Paper on AI, published in February, which is now open to public consultation (36).

### Transparency and Explainability

The “black box” problem is one of the major foci of AI ethics (37). Besides referring to the inherent opacity of complex machine learning algorithms such as neural networks, it is also the case that the increasing size of datasets used in developing AI for health makes explanations of the relationships between input data and outputs difficult—understanding how each of millions of variables contributes to the final output may be computationally intractable (38). Questions that may therefore follow include: How can patients give meaningful informed consent to, or clinicians advise the use of, algorithms the internal workings of which are unclear? (39) Should we be using black box algorithms in healthcare at all?

It is easy to forget that the human brain is itself a “black box,” given the ease with which we explain our own decisions via *post hoc* rationalization (40, 41). The field of medicine has therefore been accustomed to dealing with black box decision-making for millennia. Of course, part of the difference between an opaque human decision and an opaque algorithmic one is the ability to have a conversation with the former, such that the decision itself can be probed and aspects of the decision that are important to its subject better understood. This highlights an important concept that should be considered when grappling with the issue of explainability, which is the distinction between “model-centric explanations” (where the focus is on providing a complete account of how the model works), and “subject-centric explanations” (where “only” those aspects of model functioning that are relevant to the subject are considered) (42). Given that different subjects may require different types of explanation, there is a very strong argument for addressing the black box problem through thorough user/stakeholder research, and their meaningful involvement throughout the development process. Thus, rather than a blanket requirement of full explainability, smart regulatory frameworks may opt to give regard to the application of the AI tool, its intended target group, and its risk profile, with higher risk applications in more vulnerable groups necessitating deeper explanations. Nevertheless, we contend that one area of transparency should remain strictly enforced, namely that developers and healthcare system administrators make it absolutely clear to service users when an algorithm is being used to support or to independently provide decision-making.

### Health Inequalities

A systematic review found significant aversion amongst the UK public to health inequalities, particularly when such inequalities are presented in the context of socioeconomic differences (43). Thus, any suggestion that the use of AI in medicine may exacerbate existing health inequalities, for example by automating existing bias and unfairness at speed and scale, is likely to decrease trust in and acceptability of these tools. Sadly, there is evidence that this is already occurring. For example, an algorithm in widespread use in the US to determine the likely healthcare needs of a patient, and thus access to onward services, exhibits significant racial bias—in short, African-American patients needed to be significantly “sicker” than Caucasian patients to get the same score, and thus the same access to services, via this algorithm (44).

In the context of cardiac imaging, a specific source of inequality may result from the geographic distribution of these technologies. Much cardiac imaging, particularly using newer modalities such as cardiac magnetic resonance, is largely performed in higher-income countries, and even there, in centers of excellence or high-volume practices (45). This means that training datasets used in the development of AI models for the analysis of these images will suffer from a relative lack of images from patients in low- and middle-income countries. Even disadvantaged patients in high-income countries, who may not have access to the best, most expensive imaging, may be relatively underrepresented in such datasets. Such excluded groups may find that cardiac imaging AI tools developed with these unrepresentative datasets are either less accurate when applied to their cases, or are excluded altogether from the potential benefits of these technologies due to decisions around deployment and marketing by their manufacturers.

The question also remains as to whether the use of AI will create new health inequalities. For example, consumer-facing AI tools presuppose some degree of digital literacy, and their use is likely to pose a personal financial cost to an individual, given the expensive hardware that is frequently required, such as a smartphone or wearable technology. More work is needed to better understand which groups may be excluded from the benefits these technologies could bring, and to develop strategies to avoid such outcomes.

### Errors and Liability

Just like the black box problem, the question of “who is responsible when things go wrong with AI” has received a lot of attention in ethics and policy circles (46, 47). The Canadian Association of Radiologists has approached this discussion by focusing on degree of autonomy as a critical determinant. Seeing as most current applications of AI strictly define its role as assistive, including in “intended use” statements that carry regulatory weight, it is reasonable to suggest that ultimate liability for erroneous decisions such as misdiagnosis would rest with the responsible clinician. However, as the degree of autonomy increases, the degree of liability should shift toward the manufacturer, provided that the clinician can prove that they were using the AI tool exactly as intended. Another potential player is the healthcare system or institution that implemented the AI algorithm, especially if it is determined that, as with any other tool or technology, the organization has a duty to deploy it appropriately (21). However, there are concerns that difficulties in explaining algorithmic decisions (see section **Transparency and Explainability**) may translate into difficulties for patients who suffer harm in proving causation by an algorithm, regardless of the latter's autonomy (39). Thus, a *res ipsa loquitur* (“the facts speak for themselves”) approach may come to be preferred, where it is the manufacturer that has a *prima facie* case to rebut, and which has successfully been used in cases of harm caused by machinery (48).

### Ensuring the Public's Needs Are Met

Patients and members of the public have a more nuanced understanding of tasks and roles in healthcare than they are frequently given credit for. In research we commissioned, for example, we found that 45% of respondents (in a sample selected to be representative of the UK adult population) agreed that AI should be used to “help diagnose disease,” but only 17% agreed that it should be used to “take on other tasks performed by doctors and nurses,” such as breaking bad news; 63% said it should not be used for this purpose (7). Similarly, attitudes to data sharing for AI research are complex and nuanced. For example, in a workshop study conducted by the Wellcome Trust with 246 patients and HCPs, 17% of participants indicated opposition to giving commercial companies access to their data for the purposes of research. However, when data sharing was tied to the possibility of benefits from this research, 61% of the same study participants indicated they would rather share their data with commercial companies than miss out on potential positive outcomes (49). Many such studies of attitudes to data sharing exist [and the Understanding Patient Data initiative provides an excellent compendium of these studies and their major findings (50)], but two themes emerge across all of these studies as critical factors in determining readiness to share data: firstly, the importance of trust in the *institution* carrying out the research or development, and secondly, the importance of communicating potential benefits clearly.

### Regulation

In our 2018 report, we discussed the looming potential of a clash between existing healthcare regulators, and the new regulators, oversight bodies, and advisory committees being set up by governments and multinational organizations to focus on AI more generally, such as the Centre for Data Ethics and Innovation in the UK, and the EU's High Level Expert Group. As it turns out, no such clash has transpired, as newer AI-focused bodies have thus far been content to leave the realm of health and care to the more established regulators. However, this does not mean that regulatory certainty has followed. The healthcare regulatory space is crowded, and the speed of technological development means that these regulators have been undertaking an exercise of rapid capacity building, to be able to consider the potential impacts of these technologies. Furthermore, communication between these regulators needs to occur to ensure clear responsibility for all parts of the development process, and to avoid regulatory gaps. In the UK, the think tank Reform has released a series of resources that definitively map each step in developing a data-driven healthcare tool (from idea generation, through to securing data access, through to undertaking clinical research, to ascertaining regulatory compliance and post-market surveillance) to specific regulators, and lays out the requirements at each stage (51). The CEO of NHSX, UK Government unit with responsibility for setting national policy and developing best practice for NHS technology, digital and data, has acknowledged the need for better regulatory alignment (52). The very fact that such discussions are being had indicates the shift in thinking that is occurring in the health technology (healthtech) space, where rather than “software” and “apps,” more enlightened technologists are realizing that what they are creating are medical devices, with the risks and benefits inherent in any medical intervention. Having first been expressed by the Software as a Medical Device (SaMD) initiative kicked off by International Medical Device Regulators Forum (IMDRF), this culture shift has arguably reached its zenith in the EU's Medical Device Regulation 2017/745, the post-market surveillance requirements of which will be fully applicable by May 2020. The launch of a European database on medical devices (EUDAMED) in May 2022, with a much wider scope than the existing one, will mean that data on post-market surveillance of various devices, including AI tools, will be publicly available to an unprecedented degree.

Another area where regulators may contribute is in the development of standardized benchmarks to allow replicable assessment of the performance of AI tools, both over time and in comparison to one another. This is precisely the aim of the AI for Health (AI4H) Focus Group, a joint initiative of the World Health Organization and the International Telecommunications Union (53).

### Consequences of Novel Insights

We have already alluded to the fact that the novel methods of data analysis these AI tools could provide can lead to unexpected insights being obtained from datasets (see section Data). Taken one step further, we can envisage a situation where these tools could potentially present patients and members of the public with information that (a) would not have been previously available, and (b) has the capacity to radically alter how they think about themselves and their health. A close analogy is genomic testing, with the new insights and attendant deep ethical questions it has forced us to consider (54). Just as with genes, if algorithmic predictions come to be equated with “destiny,” then this could lead to a perception of futility and diminishment of hope. Negative consequences could include an individual fearing that they may not have access to certain interventions, and therefore not seeking them. Moreover, not everyone would like to discover that they are at high risk of one condition or another, especially if the treatment or cure options are limited. Decisions on these questions are likely to be nuanced and vary greatly in different situations and between different patients, but they should always be taken in the context of meaningful conversations between patients and their healthcare providers, and with deep appreciation of a patient's autonomy.

On a population level, algorithmic predictions of this nature can easily translate into algorithm profiling, create new categories and subgroups within existing populations. People may be assigned to these groups, and inferences and choices made about them, possibly without their knowledge (55). It is unclear where the balance should be struck between capitalizing on the new insights these algorithms could provide, and the threats to autonomy and individuality that categorization of societies and communities could lead to. An interesting suggestion has been to invoke the concept of solidarity as a means to reinforce the community-based nature of healthcare, and underlining the importance of the pursuit of a collective “good” (56).

### Trusting Algorithms

As referred to earlier (see section Ensuring the Public's Needs Are Met), trust in data-driven technologies and in their development may be intimately related to trust in the institutions responsible for this development. Further evidence for this is provided by a survey of 2000 people across Europe carried out by the Open Data Institute, where 94% of respondents said that whether or not they trust the organization asking for their data is important in considering whether or not to share data (57). It follows, therefore, to ask what it is that makes organizations trusted, and there is evidence to suggest that a major factor in determining this trust is the degree of perceived openness. Being open reduces the sense that a system or process has been captured by a particular organization or body that may not have the system's users' best interests at heart (58, 59). Moreover, openness allows the organization to demonstrate its competence in data handling, and to share its motivations for doing so; both these factors have also been found to be important determinants of readiness to engage by a systematic review (60). In order to address the requirements for openness and transparency in clinical trials involving AI algorithms, an international project is underway that aims to develop AI extensions to the existing CONSORT and SPIRIT checklists and guidance documents (61). On the other hand, given that a lot of development of AI for healthcare occurs in the private sector (see next section), legitimate concerns remain on the part of developers that regulators mandating excessive openness pose a threat to their intellectual property, and thus reduce the incentives for investment in developing these data-driven tools.

### Collaborations Between Public and Private Sector Organizations

The development of AI tools for widespread clinical use is dominated by partnerships between health and research institutions such as hospitals and universities, and private sector organizations. In the UK, there is a perception that such partnerships are needed as the healthcare system, the National Health Service, controls access to data, whereas capital for investment in R&D and the human talent required to create these tools is increasingly being concentrated in technology companies (62). There is the additional complicating factor of ensuring value not only for the patients whose data is used to develop these tools, but also for the taxpayer who funds the health service that acts as the data custodian, but who may never be in a position to directly benefit from the algorithms that are derived from such partnerships. There have already been some policy responses to such challenges. For example, following its launch in July 2019, one of NHSX's first acts was to confirm and take responsibility for enforcing a ban on exclusive data-sharing agreements between hospitals and commercial companies (63). This move has been seen as addressing concerns that exclusivity deals signed in the past by NHS hospital did not represent good value for money, and as signaling a shift toward more national decision-making on data use for technological applications.

## Developing Principles, And Translating Them Into Policy

In some countries, the response to questions such as those posed by our 2018 report has been to develop frameworks outlining principles for the ethical use of data and AI in healthcare. At the time of writing, for example, the Royal Australian and New Zealand College of Radiologists has an open consultation on its Draft Standards of Practice for Artificial Intelligence; this will close on 29th November 2019 (64). Perhaps one of the more mature frameworks is the UK Department of Health and Social Care (DHSC)'s “Code of Conduct for data-driven health and care technology,” which was developed using a Delphi methodology and was first published in September 2018. It is already in its third iteration following a process of expert review and public consultation (65). This principles-based document has been broadly well-received, and constitutes a world-first that is likely to serve as a global standard.

Nevertheless, it has been clear for some time that principles are a necessary but not sufficient condition to ensure safe and ethical development of healthtech tools. Specifically, it was realized that developers, predominantly coming from a technological background and therefore not imbued in the cultural norms and expectations specific to healthcare, needed support with demonstrating adherence to the principles laid out in documents such as the Code of Conduct. Put another way, if the Code of Conduct laid out *what* developers should aspire to, what they wanted was guidance on *how* to do it. It is on this background that in October 2019, NHSX launched a series of resources specifically aimed at addressing this question (66). This combination of principles and policy has been termed a “principled proportionate governance” model. Future Advocacy contributed to the development of this policy document by focusing specifically on Principle 7 of the Code of Conduct, which is concerned with transparency, openness, and ensuring safe integration of algorithms in existing healthcare systems. In order to address these issues, we signpost a number of existing resources that developers can use to demonstrate adherence to this principle, and classify them according to whether they are general processes that apply across all aspects of principle 7, or recommendations for specific processes that apply to certain subsections. For example, in order to conduct a meaningful, useful, and relevant stakeholder analysis, we encourage the use of value and consequence matrices in the context of the SUM principles developed by the Alan Turing Institute (67). Likewise, in order to encourage transparency around the means of collecting, storing, using and sharing data, we recommend the use of the Open Data Institute's “Data Ethics Canvas,” a freely-available resource from a highly-respected institution (68). What is apparent is that rather than attempting to reinvent the wheel, HCPs and technologists collaborating on the creation of data-driven tools for healthcare need to develop greater familiarity with the work that is already ongoing in the wider technology ethics and policy community, as this cross-disciplinary approach is likely to suggest solutions to problems the field of healthcare is only beginning to grapple with.

## Safe And Acceptable: The Future Of Ai In Health And Care

Two specific themes that run through the Code of Conduct, and that have been referred to at multiple points in this review are those of stakeholder engagement, and openness. Both these concepts are important when thinking about how developers can increase the likelihood of their tools being acceptable to patients and HCPs. For example, research with patients and members of the public has indicated that they do not want the development of these tools to come at the expense of the relationships that characterize good care (5, 7). Undertaking a robust and inclusive process of stakeholder analysis will help highlight relationships of importance in healthcare, and will ensure that the participants in these relationships involved in the development process. Furthermore, if development is guided by a deep understanding of the needs of the prospective user from an early stage, the product that comes out of the development process is more likely to be adopted and deployed. Similarly, as has been discussed previously, openness is a determinant of trust, which is itself a determinant of likelihood to engage with the development of these tools. It is therefore our recommendation that the principles of stakeholder engagement and openness run through the development of AI tools for all applications in health and care.

Although the principles and policies discussed above inspired by a drive to increase the safety of AI technologies as applied to health, they are not in themselves sufficient to guarantee safety. A detailed treatment of the various processes and standards related to the safety of these products is beyond the scope of this review, but it is noted that the shift in thinking toward treating these tools as medical devices, as described earlier and as encapsulated in the Medical Device Regulation, should go some way toward protecting users and patients, by placing more stringent requirements in terms of external audit, of developing and maintaining robust quality management systems, and of being more responsive to user feedback and field surveillance (69). Other safety issues that remain relatively unaddressed by current regulation and HCP training programmes include those of automation complacency, and of the use of dynamic, continuously learning systems (70).

## Conclusion

In this review we have updated the ten challenges we originally identified in 2018 with current thinking and practice, reflecting the rapid changes in the field of AI as applied to health and care. There is still some way to go in addressing these questions. It is clear to us that, given the iterative nature of technological development, the development of pathways for continuous review of principles and policy frameworks should be prioritized by governments and healthcare authorities. Furthermore, given the complexity of these technologies, a truly multidisciplinary approach is required. It is only by involving all stakeholders with a sincere desire to ensure the successful development and deployment of these tools that their risks will be minimized, and their opportunities maximized.

## Author Contributions

MF conducted the original research by Future Advocacy cited in this review, and wrote this paper. OB also conducted the original research cited, reviewed this paper, and provided senior approval for its publication.

### Conflict of Interest

As of 1st September 2019, MF has been employed by Ada Health GmbH, which develops AI tools for use in health applications. The remaining author declares that the research was conducted in the absence of any commercial or financial relationships that could be construed as a potential conflict of interest.
